# Experimental and numerical modeling approach for thermomechanical low cycle fatigue analysis of cyclically non-stabilized steels

**DOI:** 10.1016/j.mex.2021.101213

**Published:** 2021-01-18

**Authors:** Władysław Egner, Piotr Sulich, Stanisław Mroziński, Michał Piotrowski, Halina Egner

**Affiliations:** a Institute of Applied Mechanics, Faculty of Mechanical Engineering, Cracow University of Technology, 31-864 Kraków Al. Jana Pawła II 37, Poland; b UTP University of Science and Technology, Faculty of Mechanical Engineering, 85-225 Bydgoszcz ul. Kordeckiego 20, Poland

**Keywords:** Material testing, Thermo-mechanical fatigue, Numerical implementation, Identification of parameters

## Abstract

The widely used fatigue life prediction models, such as the Coffin–Manson model or S–N curve related models are based on the assumption that the response of a material experiencing low cycle fatigue loading is stabilized during some period. However, for many materials such a stabilized state is hardly observed, and the activated mechanisms for cyclic hardening or softening depend on test conditions. In general, the selected test conditions (stress or strain control) should depend on the intended use of the obtained material data. If testing conditions do not correspond to the operation mode of the considered mechanical facilities, the above mentioned life prediction models will produce inaccurate results. Hence, selecting and identifying proper fatigue parameters, which would represent the state of a material during the whole fatigue life, is extremely important in reliability evaluation of structures.

In the case of non-stabilizing steels, the common challenges are:

• Selecting and performing a suitable set of experimental tests to recognize various aspects of the material behavior under low-cycle thermomechanical fatigue;
• Adjusting a proper constitutive modelling, reflecting the real physical phenomena taking place in the material microstructure;
• Effective numerical implementation and optimal parameter identification.

## Graphical abstract

**Figure:**
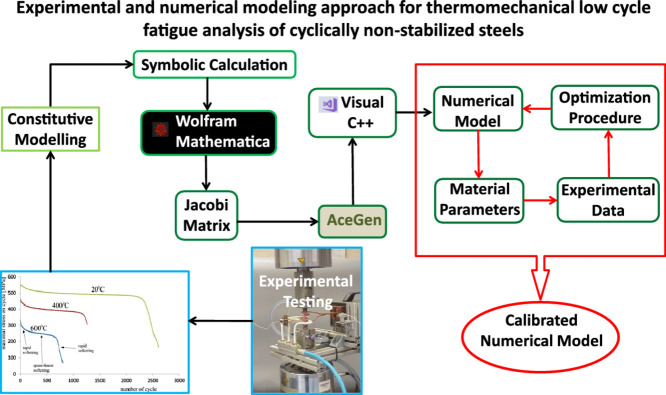

Specifications table

**Table:** 

Subject Area:	Engineering
More specific subject area:	*Fatigue*
Method name:	*Identification of thermo-mechanical fatigue behavior of cyclically non-stabilized steels*
Name and reference of original method:	*This is a co-submission. The original method is presented in:* *Egner, W., Sulich, P., Mrozinski, S., Egner, H., 2020. Modelling thermo-mechanical cyclic behavior of P91 steel. Int J Plast 135, 102820.*
Resource availability:	

## Method details

**Fig. 1:**
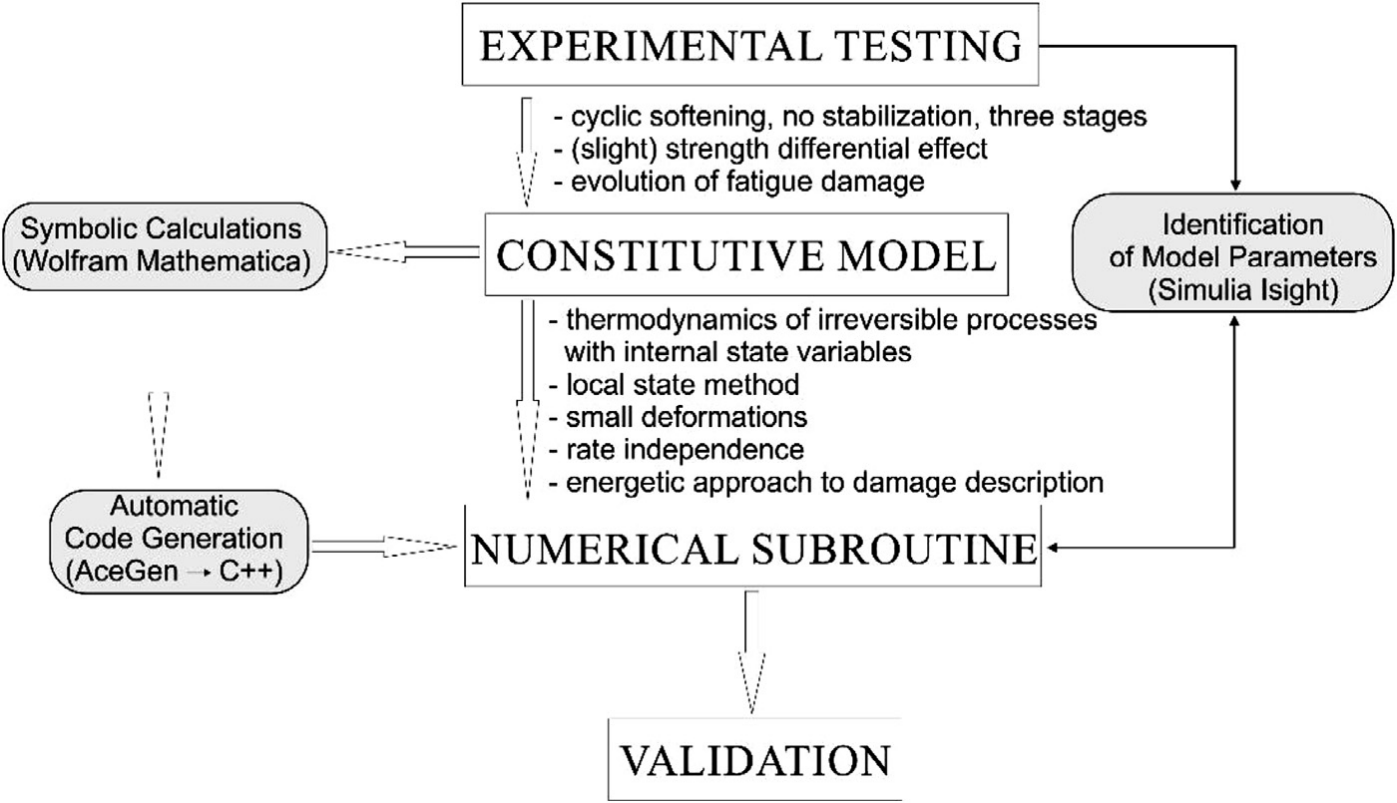
Main steps of thermo-mechanical fatigue behavior analysis.

To identify the fatigue parameters that would allow to describe a non-stabilizing steel response to the mixed stress-strain control conditions, a 4-step procedure has to be followed (see Fig. 1):

(A) The proper experimental testing has to be performed to identify different aspects of the material response
(B) A relevant constitutive model should be developed, that is capable to reflect the phenomena observed experimentally
(C) The mathematical model must then be implemented into numerical subroutines and subjected to proper parametric studies, and the optimal set of the model parameters needs to be identified on the basis of the available experimental data
(D) The modelling must finally be validated through numerical simulations of the model response to different loading conditions, compared to the experimental results.

All the equations of the constitutive model were included in the main article [4] and will not be repeated here. Instead, we present below some technical aspects of the experimental and numerical procedures which were applied to the analysis of the low cycle fatigue of P91 steel, which belongs to the class of non-stabilizing materials.

## Experimental Testing

### Test stand

The development of the energy industry puts the materials used today, working at elevated temperatures, increasingly demanding as to strength, reliability, weight, and resistance to external factors. This is due to, inter alia, the need to improve the efficiency of power plants. In many cases, this can be achieved by increasing the steam temperature used to drive the turbines. The increase in the operating temperature of power facilities causes an increase in the effort of materials used for these facilities, and thus the need to determine the strength parameters of the materials used in terms of thermal or thermo-mechanical fatigue at temperatures higher than previously used. Currently, intensive research is underway in many research centers to improve existing materials or search for new materials, as well as to research methods of these materials. It also requires improving research methods and the equipment used.

Test stands for thermal fatigue are currently technically advanced, fully computerized devices that allow controlling the course of sample temperature changes, as well as cooling and condition monitoring.

Tests in the field of thermo-mechanical fatigue may also be conducted on standard testing machines. Modern machines are most often universal devices that allow for a wide range of tests. They are characterized by very high accuracy of measurements of forces, displacements, and local deformations. Depending on the needs, test equipment can be provided with software packages dedicated to static and dynamic tests, tests in the field of brittle fracture mechanics, tests in the area of ​​low-cycle metal fatigue, and calculation of strength parameters. They not only allow to control the operation of the machine but also are used to register and then develop test results. Hydraulic strength machines enable the implementation of both simple load states (e.g. tension/compression, bending, torsion) and combined load states (e.g. simultaneous tension/compression and torsion). They can be used to test creep processes, fatigue, stress relaxation, etc. Modern endurance machines may be equipped with additional apparatus dedicated to tests in the field of thermal and mechanical fatigue: extensometers, special furnaces, diuars, interferometers, pyrometers, thermal imaging cameras, etc.

**Fig. 2:**
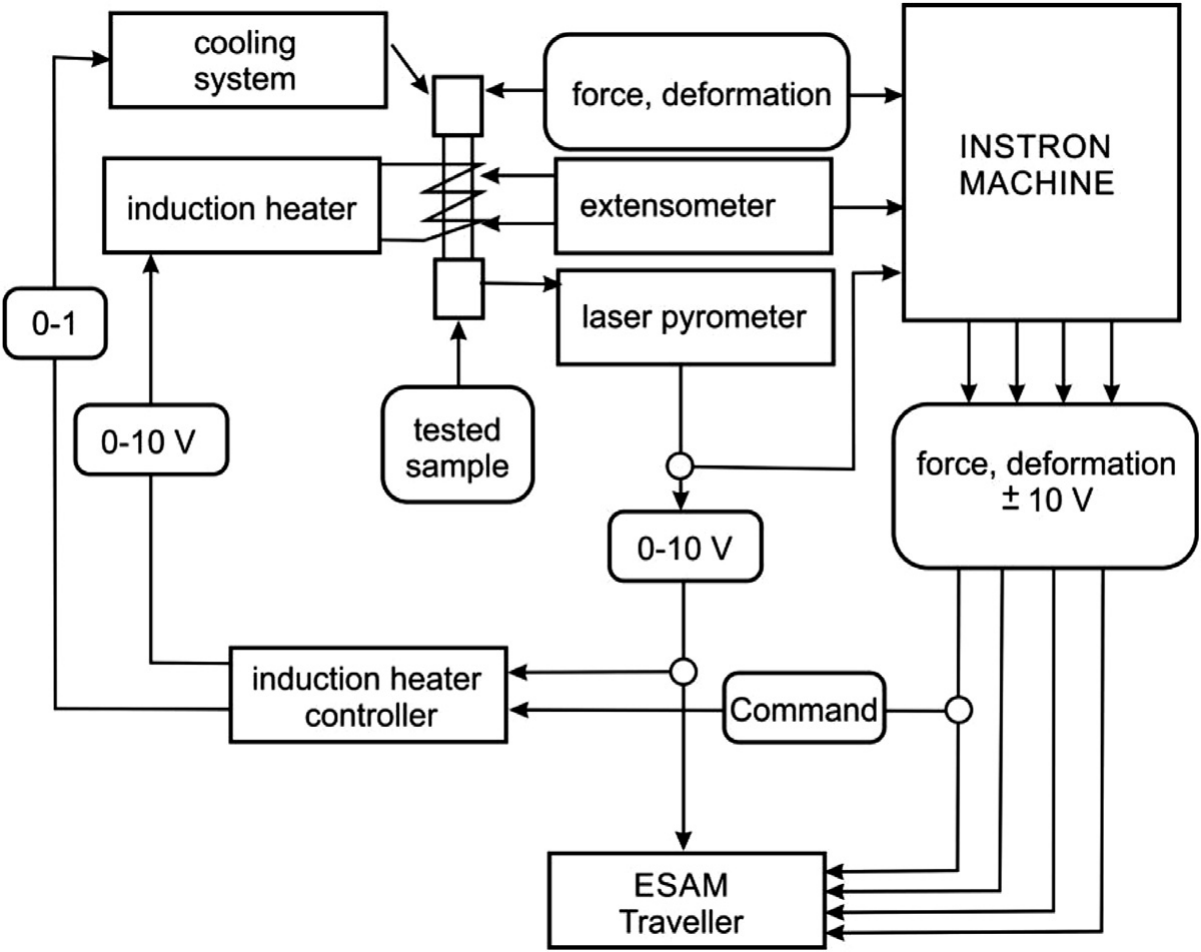
Diagram of induction heating system.

**Fig. 3:**
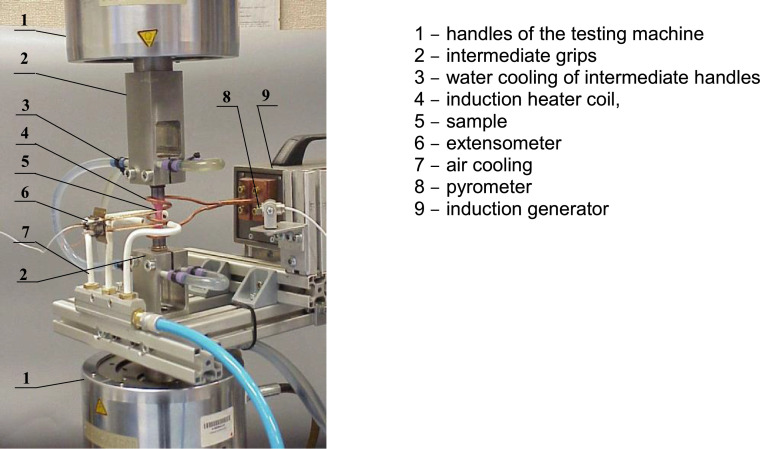
Test stand.

The implementation of fatigue tests necessary for the research described in the original paper [4] required the development of an induction heater control system and its synchronization with the testing program of a testing machine. Fig. 2 shows a diagram of the induction heating system performed, while Fig. 3 shows a view of the completed stand.

The tested sample is mounted in intermediate grips (2) of a hydraulic testing machine. The sample is heated by an induction heater through an induction coil (4). The heating efficiency depends on the power of the generator (9). For standard fatigue tests of samples with a diameter of about 10 mm, generators with a capacity of 2 to 5 kW are sufficient. The temperature of the tested sample is measured with a laser pyrometer (8). The signal from the pyrometer is transmitted to three devices: the control and measuring computer of the testing machine, multi-channel measuring bridge (ESAM Traveller) and the induction heater controller. The controller program, depending on the control variable, adjusts the heater power to the set temperature and controls the cooling system. Instantaneous values ​​of piston displacement, force, deformation, and the COMMAND control signal are recorded in the ESAM traveller multi-channel measuring bridge simultaneously with the temperature signal.

### Induction heating

Induction heating was used in the experiment because it has many advantages over other heating methods, such as chamber heating or resistance heating. The physical phenomenon used is magnetic induction. It involves the creation of eddy currents in metals subjected to an alternating magnetic field, produced by the heater coil. The thermal effect is obtained as a result of changing the energy of the electromagnetic field into thermal energy emitted inside the heated material. This feature allows heating of the measuring part of the tested sample without heating the fastening elements. The main advantage of the method is the heating rate, incomparably higher than heating in the heating chamber. This feature is particularly desirable when testing steel samples under variable temperature conditions.

Induction heating also does not require electrical insulation between the sample and the testing machine, which greatly simplifies the installation method. However, this heating method has several disadvantages. Some restrictions are caused by the generator coil, which limits access to the sample surface and fixing measuring instruments such as extensometer or pyrometer on it. Due to the high current flowing through the generator's working coil, it is necessary to cool it. This is done by flowing the appropriate coolant (air or water) through the generator coil. The disadvantages of the method also include a strong, fast-changing electromagnetic field near the induction coil. In high frequency magnetic fields, the induced current flows in the surface layer of the sample. When larger samples are heated, this results in longer heating time. In this case, it is advisable to reduce the excitation frequency. Another solution often used in practice is the use of hollow samples for testing. The use of hollow samples allows for providing a small temperature gradient while allowing the use of the internal cooling of the sample. The coolant is usually the air flowing through the hollow sample and, additionally, an external stream of air fed from symmetrically located nozzles. This solution was proposed, among others, in Evans et al. [5].

### Experimental verification of the test stand – thermal fatigue

**Fig. 4:**
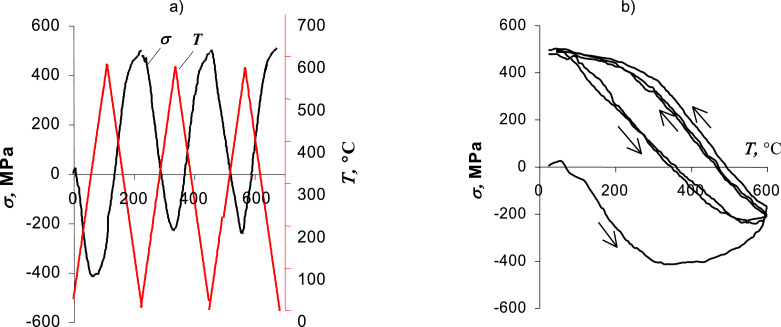
Thermal fatigue test results: a) temperature and stress changes over time, b) changes in stress as a function of temperature.

**Fig. 5:**
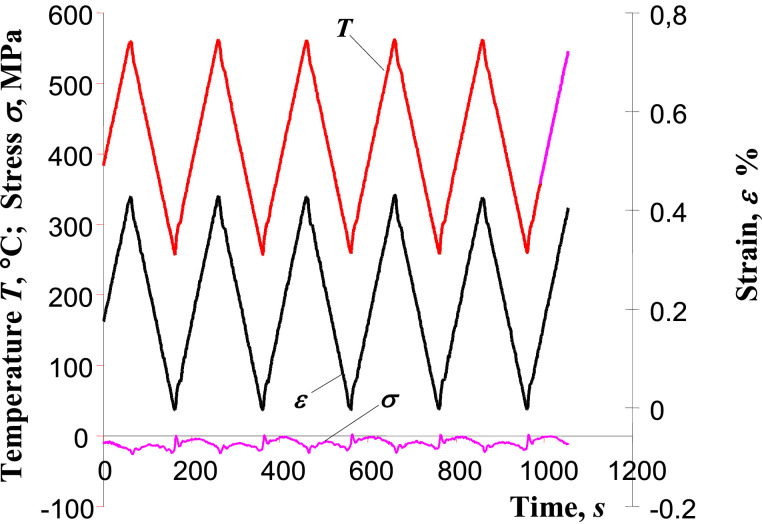
Results of zero stress test.

Thermal fatigue tests were carried out with the use of a standard Instron 8501 testing machine together with the induction heating device shown in Fig. 3. Samples made of P91 steel were used in the tests. Changes in the temperature of the restrained sample (Fig. 4a) cause compressive or tensile stresses in the sample. Fig. 5 shows the recorded deformation plots versus temperature changes. Based on the course of the stress changes in time, it can be stated that up to about 400°C these changes are linear. Above this level, the sample material is subjected to plastic slips and the relationship between stress and temperature is no longer linear. Fig. 4b presents the changes in stress during a few cycles of temperature. The application of cyclic temperature changes may, similar to mechanical loading, lead to cracks. However, the lifetime may be significantly longer.

### Experimental verification of the test stand – thermomechanical fatigue

Experimental verification of test stand control systems was conducted in the form of so-called “zero stress test”. The test consists in controlling the deformation course and temperature of the mounted sample (in-phase cycles) to achieve close stress in the sample to zero. Fig. 5 shows the results of the zero stress test of P91 steel sample. The period of temperature and strain changes was 200 s.

**Fig. 6:**
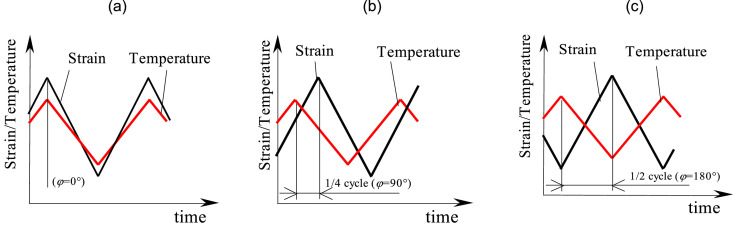
Load configurations: (a) in-phase load (*φ*=0°); (b) arbitrary phase configuration (*φ*=90°); (c) out-of-phase load (*φ*=180°).

The diagram shown in Fig. 6a corresponds to the load variant in which the maximum values of temperature and deformation occur at the same time (*φ*=0°). In Fig. 6b, the maximum value of temperature and deformation are shifted relative to each other by a time corresponding to 1/4 cycle (*φ*=90°), while in Fig. 6c the shift is 1/2 cycle (*φ*=180°).

Based on the stress graph (σ) it can be stated that despite the significant changes in sample temperature (*T*) and the associated strain (*ε*) due to thermal expansion, the stresses in the sample remained small. They may result from inaccurate determination of the thermal expansion coefficient and inertia of the control system The largest differences (about 20 MPa) occurred at the points of the temperature reversed changes. This is related to the adopted control method that makes the temperature to change in accordance with the deformation. The thermal inertia of the sample and the inertia of the heating system produce small shifts of force with respect to temperature and, consequently, small stresses in the sample.

**Fig. 7:**
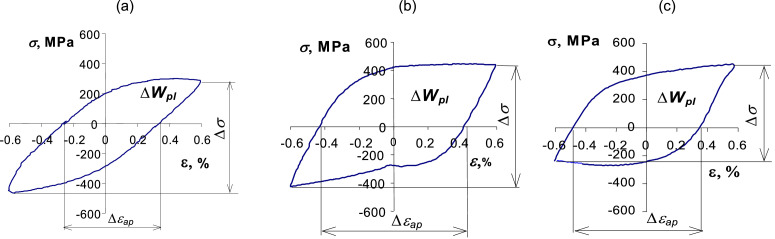
Exemplary hysteresis loops for *ε_ac_* = 0,6% and *T*_min_ =260 °C, *T*_max_ = 600 °C: (a) in-phase load (*φ*=*0°*); (b) arbitrary phase configuration (*φ*=90°); (c) out-of-phase load (*φ*=180°).

Constant-amplitude, non-isothermal tests were conducted under controlled deformation conditions (*ε_ac_* = *const*). Fig. 7 shows examples of hysteresis loops obtained at one deformation level (*ε_ac_* = 0.6%) for three load configurations shown in Fig. 6.

The hysteresis loops presented in the above diagrams were obtained at one level of strain amplitude (*ε_ac_* = 0.6%). They are characterized by the diversity of several basic parameters (plastic strain increment Δ*ε_ap_*, stress increment Δ*σ*, dissipated energy Δ*W_pl_*). In order to illustrate the impact of the load variant on these parameters, Table 1 summarizes their values at the considered level of deformation (*ε_ac_* = 0.6%).

**Table 1:** Loop parameters at *ε_ac_* = 0,6% for different load variants

Parameter	In-phase load *φ*=0° (Fig. 7a)	Arbitrary phase *φ*=90° (Fig. 7b)	Out-of-phase load *φ*=180° (Fig. 7c)
Δ*ε_ap_*, %	0,5876	0,8362	0,8321
Δ*σ*, MPa	763,6	871,5	724,6
Δ*W*_pl_, MJ/m^3^	3,875	6,159	5,084

Based on the results summarized in Table 1, it can be concluded that the load configuration affects the basic parameters of the hysteresis loop (Δ*ε_ap_*, Δ*σ*, Δ*W_pl_*). The values of these parameters have a direct impact on the fatigue life at a given strain level. They can also be used for damage accumulation carried out during fatigue life calculations. In the analyzed case, regardless of the adopted description of fatigue (stress, strain, energy), the most unfavorable configuration of load for fatigue durability is the arbitrary phase configuration (Fig. 6b). The values of the considered loop parameters are the largest in this case. The lowest values of the loop parameters take place during the in-phase course (Fig. 7b).

Due to the mutual interactions between deformation and temperature during the tests, the shift (*φ*) of the deformation and temperature affects the basic parameters of the hysteresis loop (Δε*_ap_*, Δ*σ*, Δ*W_pl_*) that have a direct impact on fatigue life on a given deformation level. In the analyzed case, regardless of the adopted description of fatigue (stress, strain, energy), the most unfavorable load course in terms of the fatigue life is the course in which the deformation and temperature are shifted by 1/4 cycle (*φ****=***90°, Fig 7b). The values of loop parameters such as Δε*_ap_*, Δ*σ*, Δ*W_pl_* are the largest during this run. The smallest values of loop parameters occur for the in-phase case (Fig. 7a).

### Test method in relation to test standards

In the standard ASTM-E2368 [1], among others, the total deformation *ε_ac_* was proposed as the controlling quantity. The research stand used in the present work (Fig. 3) additionally allows the research to be carried out under conditions *σ_a_* =const, and Δ*W_pl_* =const. For the materials that do not exhibit a stabilization of cyclic properties, a particularly significant and important parameter is the energy, Δ*W_pl_*   [4]. Low-cycle tests under the conditions of controlled plastic deformation energy Δ*W_pl_* are presented, among others, in the paper [8]. This parameter can be successfully used when summing fatigue damage in terms of energy. The above issue is discussed in more detail in the paper [9].

**Fig. 8:**
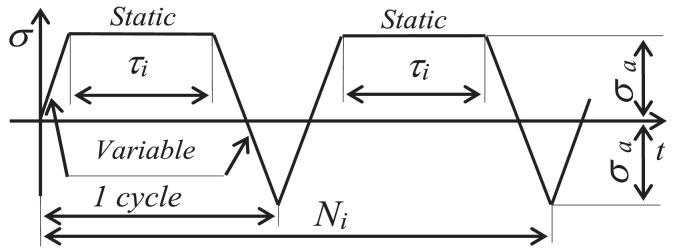
Programmable load scheme.

The standard ASTM-E2368 provides for the realization of control quantity *ε_ac_* amplitude runs being constant, while the test stand proposed in the present research additionally allows for the implementation of loads under programmed conditions. During such tests, in the load program, in addition to the cycles of variable load, creeping may also be used (Fig. 8).

**Fig. 9:**
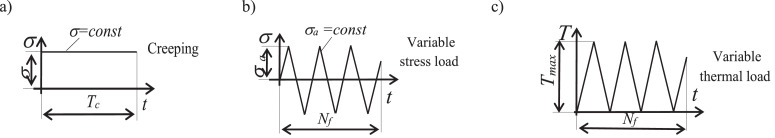
Tests that can be performed on the test stand: a) creeping, b) variable stress load, c) variable thermal load.

The stand also allows for the implementation of the following options: a static load (creeping) only - Fig. 9a, the stress variable load only (isothermal fatigue tests) - Fig. 9b, and the variable thermal load only (non-isothermal fatigue tests) - Fig. 9c.

The key issue in fatigue testing is to determine the number of cycles corresponding to the end of the fatigue test. This problem is also signaled in the standard ASTM-E2368. However, the criteria for the end of fatigue tests proposed in the standard have not proved to be successful in the research conducted by the authors. To develop the results of fatigue tests, a proprietary method of defining the end of the test was proposed. The method is described in [10]. The method complements the methods described, inter alia, in the standard ASTM-E2368.

## Constitutive Model

To reflect the observed material behavior (elastic-plastic damage material exhibiting mixed hardening) in a mathematical model, the formalism of thermodynamics of irreversible processes with internal state variables was applied. The Frederick and Armstrong [6] type constitutive model served as the basic description subjected to several extensions and modifications. To take into account the non-saturating cyclic softening observed experimentally in the case of P91 steel, the drag stress was decomposed into two non-linear parts. The first part corresponds to the strong softening (typically taking place during the first hundred cycles), while the second one allows reflecting the continuous softening. To correctly reproduce cyclic loading the kinematic type of hardening, proposed initially by Armstrong and Frederick and extended by Chaboche, was used. The thermodynamic forces conjugated to state variables result from the assumed form of the state potential, which is the Helmholtz free energy, decomposed into thermo-elastic-damage and thermo-plastic parts. To establish the rate laws the potential approach was applied, based on the assumption of the existence of dissipation potential, composed of plastic and damage parts. The kinetic equations were obtained with the use of the generalized normality rule [3]. The influence of fatigue damage was modeled in two ways. First, the classical ductile damage model [7] was applied. Motivated by the experimental observations of the influence of testing conditions on the stress- or strain-based characteristics, a dissipation-based approach to fatigue damage description [2] was also implemented for comparison.

## Numerical Algorithm

The constitutive model was implemented into numerical subroutines by the use of the fully implicit backward Euler scheme (which is always stable and very accurate) and the Newton-Raphson method. The iterative solution procedure is defined as:

$$\Delta U^{\left(k+1\right)}=\Delta U^{\left(k\right)}-{\left[J^{\left(k\right)}\right]}^{-1}{R^{res}}^{\left(k\right)}$$

where ΔU={Δɛ,Δα,Δr,ΔD} represents the vector containing the increments of the unknowns: respectively the strain tensor, kinematic hardening variable, isotropic hardening variable, and damage parameter. The residual vector $R^{res}=\left\{R_{\Delta \varepsilon },R_{\Delta \alpha },R_{\Delta r},R_{\Delta D}\right\}$ contains the differences between the respective variables $\Delta U_{i}$ and functions $\Delta {\hat{U}}_{i}$ resulting from the kinetic equations for i-th variable $U_{i}$. Finally, $\left[J\right]=\partial R^{res}/\partial \Delta U$ is the Jacobian matrix:

$$J\left(R^{res}\right)=\left[\begin{array}{cccc} \frac{\partial R_{\Delta \varepsilon }}{\partial {ɛ}^{E}} & \frac{\partial R_{\Delta \varepsilon }}{\partial \alpha } & \frac{\partial R_{\Delta \varepsilon }}{\partial r} & \frac{\partial R_{\Delta \varepsilon }}{\partial D}\\ \frac{\partial R_{\Delta \alpha }}{\partial {ɛ}^{E}} & \frac{\partial R_{\Delta \alpha }}{\partial \alpha } & \frac{\partial R_{\Delta \alpha }}{\partial r} & \frac{\partial R_{\Delta \alpha }}{\partial D}\\ \frac{\partial R_{\Delta r}}{\partial {ɛ}^{E}} & \frac{\partial R_{\Delta r}}{\partial \alpha } & \frac{\partial R_{\Delta r}}{\partial r} & \frac{\partial R_{\Delta r}}{\partial D}\\ \frac{\partial R_{\Delta D}}{\partial {ɛ}^{E}} & \frac{\partial R_{\Delta D}}{\partial \alpha } & \frac{\partial R_{\Delta D}}{\partial r} & \frac{\partial R_{\Delta D}}{\partial D} \end{array}\right]$$

The condition $R^{res}\left(\Delta U\right)=0$ defines the solution, therefore the iteration procedure is stopped when the norm of $R^{res}$ is sufficiently small.

**Fig. 10:**
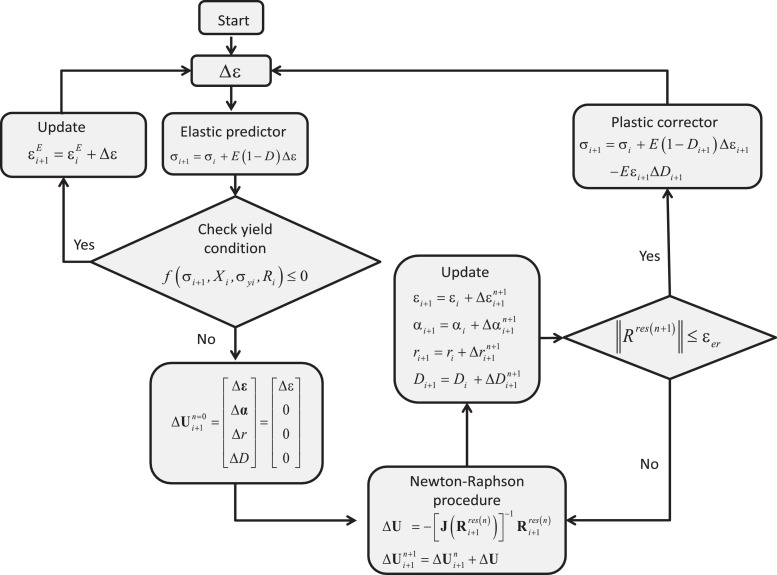
Numerical algorithm based on predictor-corrector scheme.

To solve the problem numerically, the classical concept of elastic predictor/plastic corrector was applied, see Fig. 10.

## Numerical Research Tools

### Symbolic calculations with Wolfram Mathematica

To perform calculations in the symbolic range, the Wolfram Mathematica 11.2 [12] package was used. An example of calculating the components of the plastic strain increment tensor with the use of Wolfram Mathematica 11.2 syntax is presented below.

• Generating the stress tensor:
  $$\left(\begin{array}{ccc} \sigma_{1,1} & \sigma_{1,2} & \sigma_{1,3}\\ \sigma_{2,1} & \sigma_{2,2} & \sigma_{2,3}\\ \sigma_{3,1} & \sigma_{3,2} & \sigma_{3,3} \end{array}\right)$$
• Generating the back stress tensor:
  $$\left(\begin{array}{ccc} x_{1,1} & x_{1,2} & x_{1,3}\\ x_{2,1} & x_{2,2} & x_{2,3}\\ x_{3,1} & x_{3,2} & x_{3,3} \end{array}\right)$$
• Generating the stress deviator:
  $$\left(\begin{array}{ccc} \frac{1}{3}\left(2\sigma_{1,1}-\sigma_{2,2}-\sigma_{3,3}\right) & \sigma_{1,2} & \sigma_{1,3}\\ \sigma_{2,1} & \frac{1}{3}\left(-\sigma_{1,1}+2\sigma_{2,2}-\sigma_{3,3}\right) & \sigma_{2,3}\\ \sigma_{3,1} & \sigma_{3,2} & \frac{1}{3}\left(-\sigma_{1,1}-\sigma_{2,2}+2\sigma_{3,3}\right) \end{array}\right)$$
• Yield function in the general coordinate system:
• Plastic strain increment tensor:

For the uniaxial stress state, the above command results in:

$$\left(\begin{array}{ccc} \frac{\Delta \lambda (-x+\sigma )}{\sqrt{{\left(x-\sigma \right)}^{2}}} & 0 & 0\\ 0 & \frac{\Delta \lambda (x-\sigma )}{2\sqrt{{\left(x-\sigma \right)}^{2}}} & 0\\ 0 & 0 & \frac{\Delta \lambda (x-\sigma )}{2\sqrt{{\left(x-\sigma \right)}^{2}}} \end{array}\right)$$

where *σ* and *x* denote the stress and back stress in the uniaxial state:

$$\left[\sigma \right]=\sigma \left[\begin{array}{ccc} 1 & 0 & 0\\ 0 & 0 & 0\\ 0 & 0 & 0 \end{array}\right];\left[x\right]=x\left[\begin{array}{ccc} 2/3 & 0 & 0\\ 0 & -1/3 & 0\\ 0 & 0 & -1/3 \end{array}\right]$$

### Automatic Code Generation in AceGen Software

To automatically generate the code for calculating the components of the Jacobi matrix, the AceGen system [11] was used, which is an extension of the Wolfram Mathematica.

The AceGen system allows to automatically generate the code, e.g. in C ++, based on formulas defined in the Mathematica program. The AceGen software introduces additional mechanisms to the Mathematica system that allow simplifying formulas in a way that leads to the generation of an optimized code.

As an example, the use of the AceGen system is presented to generate a C ++ procedure that calculates the components of one row of the Jacobi matrix: $vector=\{{\frac{\partial R_{\Delta D}}{\partial \varepsilon_{kl}^{E}}|}_{\left(1,1\right)},{\frac{\partial R_{\Delta D}}{\partial \alpha_{kl}}|}_{\left(1,1\right)},\frac{\partial R_{\Delta D}}{\partial r},\frac{\partial R_{\Delta D}}{\partial D}\}$.

• Define the Lamé parameters in the Mathematica system, as functions of Hooke's parameters:
• Define the elasticity tensor in the Mathematica system:
• Load the AceGen set of procedures into the Mathematica system:**<<AceGen′;**
• Indicate the file name (including the path) to which the procedure will be saved and the type of programming language:
• Define the header (prototype) of the procedure in C ++:

The input parameters to the procedure are (cf [4]): $\left\{\varepsilon^{E},E,q,s^{D},\Delta r^{p},\alpha ,\nu \right\}$, all passed by value.

The output parameter is the following vector: $\left\{{\frac{\partial R_{\Delta D}}{\partial \varepsilon_{kl}^{E}}|}_{\left(1,1\right)},{\frac{\partial R_{\Delta D}}{\partial \alpha_{kl}}|}_{\left(1,1\right)},\frac{\partial R_{\Delta D}}{\partial r^{p}},\frac{\partial R_{\Delta D}}{\partial D}\right\}$ (passed by reference).

• Define local variables and assigning them initial values, which are the values of current parameters passed to the procedure in the form of input data:
• Define fictive variables, elastic strain tensor, kinematic hardening tensor and damage increment (fictive variables are used as temporary variables to perform various algebraic operations symbolically on AceGen generated expressions, for ex. differentiation):
• Define damage-conjugated thermodynamic force:
• Define damage residuum:
• Define rules that simplify the general state of stress to the uniaxial state, with the simultaneous assignment to the tensor of fictitious variables the initial values, which are the values of current parameters passed to the procedure in the form of input data:
• Compute the results (for the general state of stress):
• Compute the results (for the uniaxial state), with the use of the above simplifying rules:
• Assign the results to variables, which are the output parameters of the procedure:
• Save the procedure:**SMSWrite[];**

The numerical procedure generated by AceGen is as follows:

The generated procedure is very simple, as the result of reducing the general state of stress to the uniaxial state of stress. However, the reduction cannot be performed before the differentiation operation is carried out.

An additional advantage of using the AceGen system is having a ready-made code generator, which can be easily modified when changing the constitutive model. We find it a really powerful tool that improves the process of implementing the constitutive model into numerical procedures.

### Identification of parameters with SIMULIA Isight package

**Fig. 11:**
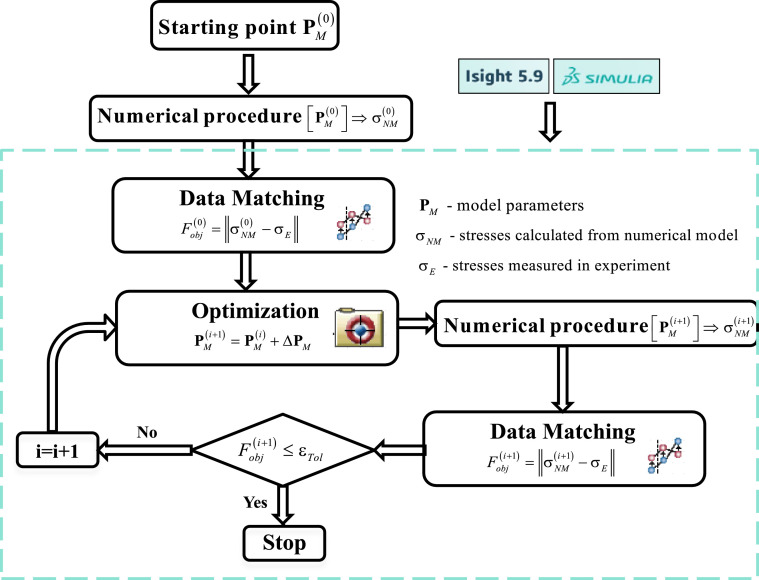
Algorithm of parameter identification with the use of SIMULIA Isight software.

In the fatigue problems, the identification of model parameters is an arduous process, the time consumption and calculation effort of which is closely related to the choice of the starting point for searching the hyperspace of parameters to find an optimal (related to the global minimum of error) parameter set. To estimate a starting point close to the optimal solution, the following steps were followed: (1) the Young modulus and yield stress were initially identified. The Young modulus was roughly determined based on the first unloading line, and the yield stress was estimated manually as an indicative point of clear curvature on the monotonic stretching part of the first hysteresis loop. (2) In the second step, kinematic plastic hardening parameters were estimated from the first loop. For the purpose of starting point estimation, it was assumed that the Young modulus and yield stress were kept constant, the parameters of isotropic hardening were zeroed and damage development was inactive. Turning off the isotropic hardening mechanisms is justified by the fact that the drag stress is proportional to the cumulative plastic strain, which is relatively small in the first cycle. (3) In the third step, the parameters of isotropic hardening were estimated while the Young modulus, yield strength, and kinematic hardening were kept constant. (4) In the last step, parameters related to damage development were estimated, with already determined values ​​of other parameters. Such initial parameter estimation allowed to set a starting point for the actual optimization of the material parameters. The approximate initial values of model parameters established in steps (1)-(4) described above, are then subjected to full optimization without any assumptions, in particular without assuming that isotropic hardening in the first cycle is turned off. The identification was carried out using the SIMULIA-Isight software [13] (see Fig. 11). The program includes the ``Data Matching'' component that automatically compares different data sets, and calculates various error measures. The ``Optimization'' component contains a variety of methods of searching for the optimal solution. In the present research, the most commonly used was the method called ``Pointer Automatic Optimizer'', based on the following optimization methods: (a) evolutionary algorithm, (b) the Nelder and Mead downhill simplex method, (3) sequential quadratic programming (NPQL), and (4) Tabu Search method. A hybrid combination of these methods appears to solve a broad range of design optimization problems.

## Declaration of Competing Interest

The Authors confirm that there are no conflicts of interest.
